# Integrating wearable devices into perioperative medicine: The potential, and future challenges

**DOI:** 10.1016/j.fhj.2024.100169

**Published:** 2024-08-08

**Authors:** Alexander Hunter

**Affiliations:** Department of Anaesthesia, University Hospitals Plymouth, Derriford Road, Plymouth, PL6 8DH, United Kingdom

## Introduction: the setting

In the UK NHS, approximately 2,400 major elective surgical procedures per 100,000 population per annum are undertaken. As demand for surgical procedures increases and patients become more complex, older and have multiple health conditions, a key priority is to provide individualised care to keep complications, which are costly to both for the patient and service provider, at a minimum. To provide this care, risk stratification is an important tool that allows us to tailor perioperative interventions to the requirements of the individual patient. A cornerstone of this risk stratification over the last decade has been routine use of cardiopulmonary exercise testing (CPET) prior to major surgery.^1^ This test aims to replicate the significant physiological strain evoked by major surgery. There is consistent evidence that preoperative aerobic capacity predicts adverse perioperative outcomes across multiple surgical settings.^2^^,^^3^ It follows that preoperative improvements in aerobic capacity through ‘prehabilitation’ should increase patient tolerance of the surgical insult and thereby reduce perioperative risk.^4^

Moreover, multidimensional prehabilitation, leveraging the surgical encounter as a ‘teachable moment’ can allow patients to adopt risk-reducing health behaviours^5^ longer term. These combined behavioural interventions are becoming commonplace in preparing patients for surgery, albeit with limited evidence of their outcomes. The prospect of establishing sustained health behavioural change through perioperative medicine is alluring; however, there are a number of challenges facing the current perioperative model.

CPET testing is time-consuming, costly and few centres have capacity to test all those who might benefit. CPET evaluates a sub-maximal and peak exercise response but provides limited assessment of a patient's behavioural exercise pattern, required for effective rehabilitation. It provides a static assessment without dynamic feedback, and it is well-accepted that patient fitness may change between assessment and surgery. Patients in need of prehabilitation are typically identified through CPET testing, but the capacity and resources to determine their prehabilitation compliance or retest patients following prehabilitation is usually lacking. Furthermore, prehabilitation plans have a series of common issues, including assessment of baseline activity, monitoring adherence with any intervention and maintaining follow-up. Studying a prehab intervention is also challenging, labour-intensive and costly. As a result, the majority of studies in this area are limited to small cohorts, typically <100 patients.

## The digital potential

Widely available consumer wearable or smartphone-based activity monitors provide a number of potential solutions to the aforementioned issues and come at a time when app-based and remote monitoring devices are increasingly familiar and acceptable to the perioperative population. They provide a method to manage preassessment, prehabilitation and rehabilitation within the perioperative population on a wide scale.^6^^,^^7^

Generally speaking, the majority of research questions these devices pose fall under the following headings;

1. *Can these devices be used as a tool for preoperative risk stratification*? Data from these devices may be able to; provide an alternative quantitative risk stratification strategy preoperatively,^8^ determine the requirement for preoperative CPET and predict preoperative CPET results. This may thereby assist in shared decision making, with patient, institutional and environmental benefit.
2. *Can data from these devices be used to assess postoperative outcomes?* New data collected by these devices, for example patient step count, may provide the means to independently assess patient outcome or improve the performance of currently available outcome scoring systems.
3. *Can these devices be used as a tool to monitor patients remotely and instigate behavioural change before or after surgery*? Devices can allow assessment of compliance with home-based exercise plans and observation of patient recovery. This in turn may allow structured ‘prehab’ and ‘rehab’ interventions, designed to instigate behavioural change, to be assessed.

## The challenges

Despite the potential, there are a number of challenges to integrate these devices into clinical practice and a number of considerations required when designing studies. These and some solutions are detailed below.

### Replicating the strain of surgery

For those who benefit most from CPET, typically undergoing major surgery, the procedure often represents a significant physiological strain and a very high oxygen uptake requirement for that individual. CPET clearly provides a distinct role here: it accurately assesses physiological response to a controlled, reproducible exercise test. Whereas wearable devices track patients in free-living environments with the majority of patients typically exposing themselves to an exercise dose well below that demanded by a CPET test. Therefore, although it is arguable that assessing patients habitual exercise patterns is useful (and indeed the regularity with which a patient stresses their cardiovascular system is also of merit) wearable devices lack the ability to fully replicate a CPET test and information gained is therefore distinct. It should be noted with interest, however, that although in the very early stages (see validity below), devices are beginning to make predictions of maximal oxygen uptake from heart rate and activity data,^9^ which may provide interest for future work.

### Validity of devices

This remains a substantial problem facing the field. Researchers must initially choose a device to use. Research-specific devices can have the advantage of being more accurate, validated, and data are often presented is a more readily analysable format. Consumer devices are often designed to have an improved user experience and have the advantage of being easily utilised en masse beyond the study population. However, validation often comes from lab-based settings or is just assumed. For data to be comparable between studies and devices, and recommendations rolled out at the population level, the accuracy and validity of the chosen device must be confirmed.^10^ This issue is, for the first time, detailed in recent consensus statements.^11^

Devices typically rely heavily on wrist-worn heart rate monitoring using photoplethysmography (PPG) (rather than electrocardiography, ECG). While the advantage is that these are better tolerated by the user, there are recognised limitations of PPG devices that reduce accuracy.^12^ Device data are often derived using proprietary algorithms, which again can limit their assessment in the research setting.

### Baseline data

Along with this, representative baseline population data using data from wearable devices (such as patient step count, sedentary minutes, active minutes) are not known and would be a valuable addition to the field, especially when designing a perioperative intervention. This would allow intervention design to be dictated by known population norms. It is regularly quoted that 10,000 daily steps is an accepted marker of high activity; however, the evidence base for this is limited, and certainly not applicable to the perioperative population. Study design can to some extent mitigate this issue by collecting baseline data per patient and so each patient in essence becomes their own control and hence small changes can be amplified due to the nature of statistical tests used for such analysis.

### Study design

Managing a typical perioperative cohort, who are often older, with a medical device is difficult. As such, the majority of work in this area involves small heterogenous cohorts. Defining the required power for future studies is therefore also difficult. As a study cohort grows to provide the dataset required to power studies capable of answering the key questions in this field, so too does the risk of missing data (see below), the labour required for maintaining data collection and the overall cost of delivering the study. Data collection can be improved with digital acquisition systems (outlined below) and devices can be cleaned and reused on subsequent participants in order to increase the number of recruited patients per costed study device.

Participants need to be sampled appropriately for outcome associations to be robust. Those who are more willing to consent may be younger, more tech savvy and interested in health data. Postoperative complication rates are also likely to vary within patient groups. As such, stratifying participants into groups by age and/or health condition is required when outcomes are being assessed.

The granularity of data derived from wearable devices is on a scale not routinely seen in perioperative medicine. The potential for engineering new strategies for analysis of this data is vast and the requirement for researchers with a strong background in data science and machine learning is likely to be of benefit to a research group.

### Missing data

This is an invariable problem with studying these devices. The reasons for lack of adherence are multifactorial. Studies must develop a strategy to limit missing data and define adherence to device use when data are missing. It should be noted that cross-sectional study data suggest that six days monitoring, inclusive of Saturday and Sunday, are needed to reliably capture weekly habitual activity in all activity intensities using the wrist-worn accelerometers.^13^ As such, a minimum of 7 days of data is likely to be required for analysis of wearable data.

Simple strategies such as routine telephone calls are labour intensive and become less feasible as participant numbers increase. More sophisticated solutions include digital dashboards that allow real time monitoring of participants and also allow outcome data collection. Labfront^14^ and Fitrockr^15^ platforms represent examples of such software, specific for Garmin devices. These allow participants not uploading data to be flagged up and also reminders to be sent via the device or user smartphone to encourage adherence. Though these strategies come at risk of digital exclusion, especially among older study participants. A combination of telephone, face-to-face and digital data collection strategies to reduce missing data is likely to be required.

Researchers may wish to consider methods to improve signal quality when using PPG devices. These can include appropriate fitting of the device anatomically and moving the device location, eg from wrist to bicep.

Where data are missing from either signal quality loss or non-wear, management of this in the methodology will be required. Consideration of deletion of data or imputation of missing values may be required.

### Digital exclusion

This is a significant multifaceted issue. The majority of wearable devices require a smartphone and some interaction with often previously unseen technology. The potential for exclusion based on age, digital literacy and socio-economic deprivation must be considered in study design in order to prevent worsening health inequality. Ensuring a strategy to make the wearable device as easy to use as possible and representation of socio-economically deprived groups within study recruitment are starting points to manage this issue. Supporting participants with data upload as described above is essential.

### Hawthorne effect

The concept that observing behaviour with wearable devices will modify this behaviour is nearly unavoidable in this setting despite being routinely criticised in many study methodology reviews. Partial mitigation will come from using devices in a consistent, non-judgemental way over as long a period of time as reasonably possible.

### Industry bias

Motivation from industry partners for using consumer devices in studies is high. Researchers should be open and transparent about their reasons for choosing a particular device and the funding received from industry partners. Researchers should look to seek access to raw data and signal processing algorithms where possible to further understand their impact on research and allow robust testing of these devices.

## Conclusion: the demand for further research

The potential for wearable technology to improve the care delivered to the perioperative population is clear. The future of this field is dependent on researchers designing studies that mitigate against the challenges outlined in this article where possible. Funders should reward studies designed to develop knowledge in this new and exciting field.

## Declaration of competing interest

The authors declare that they have no known competing financial interests or personal relationships that could have appeared to influence the work reported in this paper.
